# Enhancing the corrosion resistance of reinforcing steel under aggressive operational conditions using behentrimonium chloride

**DOI:** 10.1038/s41598-019-54669-y

**Published:** 2019-12-02

**Authors:** A. Bahgat Radwan, Mostafa H. Sliem, Noor S. Yusuf, Nasser A. Alnuaimi, Aboubakr M. Abdullah

**Affiliations:** 1 Center for Advanced Materials, Qatar University, Doha, P.O. Box 2713 Qatar; 2 Department of Civil and Architectural Engineering, Qatar University, Doha, P.O. Box 2713 Qatar

**Keywords:** Chemistry, Engineering, Materials science, Environmental impact

## Abstract

Aggressive operational conditions e.g. saline media and acidic gases, e.g., CO_2_ can increase the corrosion rate of reinforcing steel. Accordingly, the necessity to protect the steel under the above conditions without affecting the mechanical properties of the concrete is growing. Herein, the inhibition efficiency of a new corrosion inhibitor, behentrimonium chloride (BTC, C_25_H_54_ClN), is explored in a simulated-concrete pore solution (SCP) with 3.5 wt.% NaCl at different pH using electrochemical impedance spectroscopy (EIS) and polarization methods. Using only a 50 μmol L^−1^ of BTC, we are able to measure an inhibition efficiency of 91, 79, and 71% in SCP solution with 3.5% NaCl at pH of 12.5, 10 and 7, respectively without showing any effect on the mechanical properties on the cured mortars. Temkin isotherm is used to describe the physisorption of BTC inhibitor on the steel surface. Also, the adsorption and influence of the inhibitor on the metal surface are characterized using the scanning electron microscopy, atomic force microscopy, and X-ray photoelectron spectroscopy. In conclusion, this new inhibitor shows high corrosion inhibition efficiencies under different aggressive conditions and can be used in concrete to reduce the corrosion rate of reinforcing steel without decreasing the mechanical properties of the concrete.

## Introduction

Corrosion mitigation has attained a vast interest due to the high economic impact of replacing the damaged parts with new ones especially in reinforced concrete structures^1^. It is well-known that once corrosion starts in reinforcing steel, the rust (corrosion product) occupies two to three times more volume than the un-corroded steel. This higher volume induces pressures around the reinforcing bar and causes cracking of the surrounding concrete.

In general, a concrete solution has an alkaline nature (pH ~ 13) owing to the existence of sodium oxide (Na_2_O), and potassium oxide (K_2_O) in addition to calcium hydroxide as a result of the hydration reaction of calcium silicate hydrate in cement (CSH) with water from the surrounding environment^2–7^. Accordingly, an oxide layer is existing on the reinforcing steel surface within concrete^8–11^. However, penetrations of aggressive anions like chloride (Cl^−^) and sulfate (SO_4_^2−^) ions can lead to localized damage of the passive film which increases the corrosion rate of steel^1,8,10^. A corrosion current density (*i*_corr_), of around 0.2 μA cm^−2^, indicates active corrosion^12^, 0.1 μA cm^−2^, is safe for typical design life requirements of reinforced concrete structures^13^, while *i*_corr_, less than 0.01 μA cm^−2^, is low enough to avoid corrosion-induced cracking indefinitely^14^. Consequently, the inhibitors to be used in simulated concrete pore solution should satisfy two conditions; (i) a high inhibition efficiency in the existence of destructive ions, e.g. Cl^−^ ions, at different pH values (from 7 to 12.5) and (ii) no influence on the mechanical attributes of the concrete^8,15,16^. Abd El Haleem *et al*.^9^, used different inorganic inhibitors in saturated calcium hydroxide. The outcomes pointed out that the inhibition efficiency (*IE*%), of the inhibitors, improved in the following order MoO_4_^−2^ > WO_4_^−2^ > HPO_4_^−2^ > CrO_4_^−2^. However, the disadvantages of using inorganic inhibitors in concrete environments are their toxicity to living beings, cost, and inefficiency for localized corrosion^8,17^. Ormellese *et al*.^1^, has studied the long-term inhibition effectiveness of over 80 organic compounds from three main categories: amines and alkanoamines, amino alcohols, and carboxylates in SCP solution containing 0.01 M NaOH at pHΣ12.6 in the absence of chloride ions. The results showed an increase in the effectiveness of the inhibitors in the following order: carboxylates > amino acids > amines and alkanolamines. Abd El Haleem *et al*.^10,15^, highlighted the influence of benzotriazole (C_6_H_5_N_3_) and its derivatives, 5-nitrobenzotriazole (C_6_H_4_N_4_O_2_) and 5-chlorobenzotriazole (C_6_H_4_ClN_3_), on reinforcing steel inhibition in an SCP solution containing 1 M NaCl. It was found that the *IE*% of the explored inhibitors dwindled accordingly: 5-chlorobenzotriazole ˃ benzotriazole ˃ 5-nitrobenzotriazole. The maximum attained *IE*% was 69% in the presence of 5×10^−4^ M 5-chlorobenzotriazole. Interestingly, 0.0025% of deoxyribonucleic acid (DNA), showed *IE*% of 94% in SCP solution containing 3.5 wt. % NaCl with an increase of 3.61% in the compressive strength (Fc), after 28 days^18^. Zhang *et al*.^19^, achieved an inhibition efficiency of 83.15% using maize gluten meal extract as an ecologically friendly inhibitor for reinforcing steel in SPC containing 3.5 wt.%NaCl. The synthesized inhibitor of 4-(1-(4-methoxyphenyl) cyclohexyl)phenyl 9-oxodecanoate (MPOD) by Unnisa *et al*.^20^, exhibited and *IE*% of 71.81 in SCP solution including 0.5 M NaCl. On the other hand, the chemi-physisorped polymethacrylic acid co-acrylamide corrosion inhibitor displayed an *IE%* of 92.35 in SCP containing 1.8 wt.% chlorides^21^. Shanmugapriya *et al*.^22^, achieved an *IE*% of 98 in SCP using an aqueous extract of turmeric. Anitha *et al*.^23^, used the extract of rosa damascene leaves as nature-friendly inhibitor in SCP which achieved an *IE*% of 82. Wang *et al*.^24^, found that using 0.0008 mol L^−1^ of calcium lignosulfonate (CLS) showed a high *IE*% of 93.7 after immersion of carbon steel in SCP for 7200 h in comparsion to sodium oleate (SO), that exhibited an *IE*% of 40–60. Cao *et al*.^25^ explored the inhibition behaiour of phytic acid in carbonated concrete pore solution containing 0.6 mol L^−1^ NaCl on 20SiMn steel, which displayed an *IE%* of 84.0 after immersion for 72 h. Asaad *et al*.^26^, prepared non- poisonous corrosion inhibitor of silver nano-particles doped palm oil leaf extracts for renforcing steel in salin water. It was found that the addition of silver nanoparticles in the green inhibitor lead to increase the *IE%* to 94.7 after immersion for 365 d, owing to the presence of excess calcium silicate hydrate and the enhancement of the pore construction and therefore decrease the conductivity of the pore solution.

In this work, the effectiveness of a new inhibitor (behentrimonium chloride, C_25_H_54_ClN) for the corrosion of reinforcing steel in highly saline SPCs at ambient temperature and different pH values is explored. Behentrimonium chloride (BTC) is commonly used in hundreds of personal care products as conditioning and anti-static agents. Interestingly, Cameron *et al*.^27^, found that BTC is biologically safe for humans when used in a concentration range up to 5%. However, the European Union recently restricted its use for more than ≥ 1%. Consequently, we, for the first time, report the use of BTC as a corrosion inhibitor for reinforcing steel in saline SPC solutions of different pH values at significantly low concentrations of 2.5, 5, 10, and 20 ppm using electrochemical and surface analysis techniques.

## Experimental

**Figure 1:**
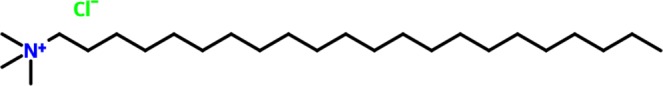
The chemical structure of behentrimonium chloride surfactant used as a corrosion inhibitor.

The reinforcing steel samples were abraded by silicon carbide grit papers using a grinding machine (Jean Wirts TG 200, Germany), sonicated with acetone, rinsed by deionized water and after that desiccate in air. The mild steel rebar contains (wt.%) C = 0.128, Si = 0.25, Mn = 0.7, Cu = 0.15, P = 0.04, S = 0.03, and rest was Fe. Saturated calcium hydroxide (Ca(OH)_2_), were used as an electrolyte to mimic SCP in 3.5 wt.% NaCl. NaCl was purchased from Sigma Aldrich and Ca(OH)_2_ from Riedel-de Haën. The pH of the SCP solution under investigation was 12.5 for the saturated Ca(OH)_2_, 10 or 7. The pH was reduced by addition of NaHCO_3_ powder to the SCP^28^. The electrochemical measurements were done at ambient temperatures using a GAMRY 3000 potentiostat/galvanostat/ZRA (Warminster, PA, USA). EIS measurements were investigated in a frequency range of 100 kHz to 0.01 Hz with an AC amplitude of 5 mV. In all electrochemical measurements, a saturated calomel electrode (SCE) and a graphite rod were employed as a reference and counter electrodes, respectively. The mild steel samples with surface areas of 0.765 cm^2^ were subjected to the SCP. All the mild steel coupons were sited under open circuit (OCP), conditions for 30 min before initiating any electrochemical test to attain the steady-state conditions. Polarization curves were attained from −0.25 to + 0.25 V against the open circuit potential (OCP), with a scan rate of 0.167 mV s^−1^. Various concentrations (0, 2.5, 5, 10, and 20 ppm) of BTC (molecular weight = 404.164 g mol^−1^), which are equivalent to 12, 24, 37 and 50 μmol L^−1^, respectively, were synthesized in the simulated saline SCP solutions. The BTC inhibitor was attained from Shanghai Dejun Chemical Technology Co., Ltd, Shanghai China, and its chemical formula is displayed in Fig. 1. Each electrochemical measurement was repeated three times to confirm the reproducibility, and the average values were reported.

### Mechanical characterization

The effect of BTC inhibitor on the compressive and flexural strength of the mortars prepared according to the ASTM C109/C109M and ASTM C348, respectively, was evaluated after different exposure times in the existence and absence of the BTC corrosion inhibitor^29,30^. The compressive experiments were performed using a 300 KN Tecnotest 3 compression testing machine (Tecnotest, Modena, Italy). The flexural strength experiments were utilized by a Lloyd LR 50 K universal testing machine (Ametec Inc. USA). The results are obtained by averaging three repeated tests. The mortar with 50 µmol L^−1^ of BTC was prepared by the mechanical mixing, in a stainless steel mixer, one part mass of cement and one and a half part mass of standard sand, with a water/cement ratio of 0.485^31^. Then, the mold was filled with the mixture under vibration to release air bubbles, and thereafter stored in a moist atmosphere for 2 days. After that, the demolding of the prepared specimens was conducted, and the samples were kept under tap water over the test period^32^. The cured mortar samples were removed from the water and located in a drying oven at 60 °C for 24 h before the strength test in order to shun the impact of the hydration of the concrete and to increase the strength of the measured samples^33^.

## Results and Discussion

### EIS

**Figure 2:**
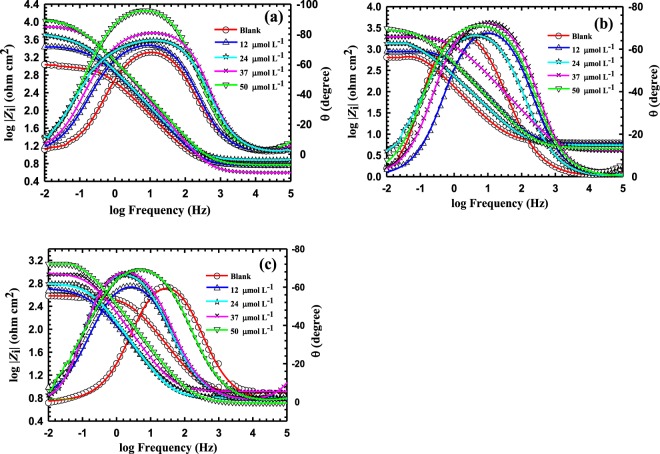
(**a**) Bode spectra for reinforcing steel in 3.5 wt.%NaCl at ambient temperature using variable concentrations of BTC inhibitor (12, 24, 37, 50 μmol L^−1^), at variable pH values of (**a**) 12.5, (**b**) 10 and (**c**) 7.

**Figure 3:**
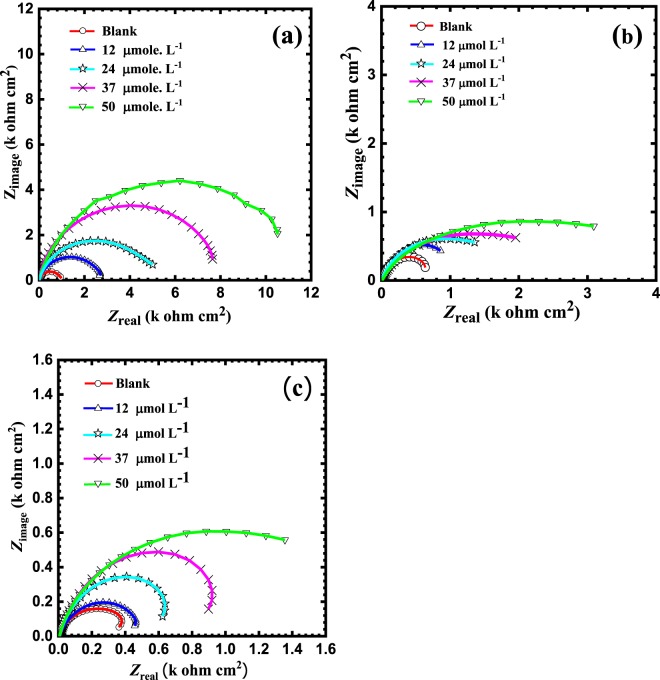
(**a**) Nyquist plots for reinforcing steel in SCP solution with 3.5 wt.%NaCl at ambient temperature using variable concentrations of BTC (12, 24, 37, 50 μmol L^−1^), at variable pH values of (**a**) 12.5, (**b**) 10 and (**c**) 7.

**Figure 4:**
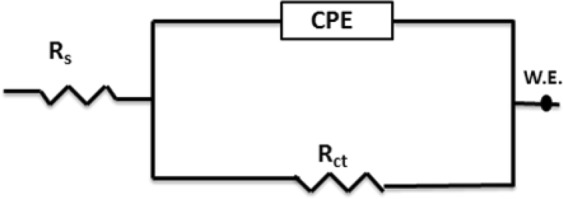
Equivalent circuit utilized to fit the EIS data for reinforcing steel in SCP containing 3.5 wt. % NaCl at variable pH.

Figures 2 and 3 display the Bode and Nyquist plots, respectively, for the reinforcing steel in SCP solutions containing 3.5 wt.%NaCl and BTC inhibitor concentrations of 12, 24, 37, 50 μmol L^−1^ at pH values of 12.5, 10 and 7 within a frequency range from 0.01 Hz to 100 k Hz at an Ac amplitude of 5 mV. Figure 4 exhibits the equivalent circuit (EC) utilized to fit the measured EIS data to obtain the different parameters that explain the metal/solution interface. The parameters are listed in Table 1 in which *R*_s_ and *R*_ct_, are credited to the electrolyte resistance and the charge transfer resistance, respectively. However, the constant phase element is expressed by (*CPE*), which is used for a non-ideal double layer. The imperfectness behavior of the double layer is accredited to the following parameters (i) a non-uniform surface coverage, (ii) surface roughness, and (iii) nonuniform current distribution or corrosion rate.

**Table 1:** Electrochemical elemen ts attained from the EIS spectra of the reinforcing steel in SCP solution with 3.5 wt.% NaCl in the existence of 12, 24, 37, 50 μmol L^−1^ of BTC corrosion inhibitor at variable pH values.

	Inhibitor concertation (µmol L^−1^)	*R*_ct_, Ω cm^2^	Y × 10^−6^ s^n^ ohm^−1^ cm^−2^	n	*C*_dl,_ × 10^−5^ F	Inhibition efficiency (*IE*%)	Surface coverage (ϴ)	Goodness of fit (χ^2^)
pH = 7	Blank	377 ± 5.3	96 ± 22	0.88 ± 0.02	6.1	—	—	2.1 × 10^−5^
	12	461 ± 42	78 ± 10	0.84 ± 0.11	4.1	18	0.18	1.5 × 10^−5^
	24	637 ± 52	70 ± 12	0.81 ± 0.08	3.4	41	0.41	3.4 × 10^−6^
	37	924 ± 28	66 ± 6	0.78 ± 0.12	3.1	59	0.59	5.2 × 10^−5^
	50	1362 ± 100	59 ± 8	0.75 ± 0.21	2.5	72	0.72	1.8 × 10^−5^
pH = 10	Blank	643 ± 18	89 ± 11	0.85 ± 0.02	5.3	—	—	6.5 × 10^−6^
	12	863 ± 24	74 ± 15	0.81 ± 0.09	3.8	25	0.25	4.2 × 10^−5^
	24	1349 ± 31	61 ± 17	0.80 ± 0.03	3.2	52	0.52	1.1 × 10^−4^
	37	1953 ± 52	50 ± 13	0.79 ± 0.05	2.7	67	0.67	2.4 × 10^−6^
	50	3105 ± 120	44 ± 28	0.72 ± 0.12	2.1	79	0.79	1.1 × 10^−5^
pH = 12.5	Blank	960 ± 66	80 ± 32	0.82 ± 0.06	4.5	—	—	8.1 × 10^−5^
	12	2678 ± 133	64 ± 9	0.75 ± 0.13	3.5	64	0.64	7.7 × 10^−6^
	24	5009 ± 149	49 ± 15	0.72 ± 0.09	2.8	80	0.80	3.6 × 10^−5^
	37	7680 ± 352	39 ± 10	0.66 ± 0.17	2.1	87	0.87	8.1 × 10^−6^
	50	10526 ± 420	28 ± 15	0.56 ± 0.27	1.1	91	0.91	1.9 × 10^−5^

The admittance and impedance of the CPE is given by^34,35^:

1 $$1/{Z}_{{\rm{CPE}}}={Y}_{{\rm{p}}}{({\rm{j}}\omega )}^{{\rm{n}}}$$

where *Z*_CPE_ is the CPE impedance (Ω cm^−2^); *Y*_p_ is the numerical value of the admittance 1/│*Z*│, at *ω* = 1 (rad s^−1^) and *j*^2^ = −1. *ω* is the angular frequency and *n* is the deviation element which varies from 0 and 1. When *n* = 1 or 0, *Z*_CPE_ is corresponding to an ideal capacitor or resistor, respectively.

The influence of the thickness and dielectric constant of the double layer is defined by the Helmholtz regime, given by the following formula:

2 $${C}_{{\rm{dl}}}=\frac{\varepsilon {\varepsilon }_{{\rm{o}}}\,A}{\delta }$$

where *Ɛ*_o_ and *Ɛ* are the dielectric constant of air and electrolyte (mainly water), respectively and *A* is the surface area of the working electrode.

The inhibition efficiency (*IE*%), is calculated using Eq. 3,

3 $$IE \% =(\frac{{R}_{{\rm{ct}}1}-{R}_{{\rm{ct}}2}}{{R}_{{\rm{ct}}1}})\times 100$$

Table 1 exhibits that the higher the corrosion inhibitor concertation is, the higher the *R*_ct_ and lower C_dl_ values are which indicates that the ability of Cl^−^ ions to attack the reinforcing steel surface declines due to the presence of a protective adsorbed layer of BTC inhibitor. It is worth to mention that lowering the pH lead to alleviating the *IE*% from 91% at pH=12.5 to 79% and 72% at pH 10 and 7, respectively. Three reasons can justify the chloride-induced loss of passivity of the reinforcing steel. First, an induced de-passivation owing to adsorption of Cl^−^ ion on the passive film at potential values higher than a critical value. Second, the penetration of Cl^−^ ions into the oxide layer leading to the formation of chloride-contaminated oxides. Finally, a mechanical film breakdown due to Cl^−^ ions adsorption which can attenuate the surface tension, thus leading to a localized disturbance in the mechanical stability of the passive layer^36^.

**Figure 5:**
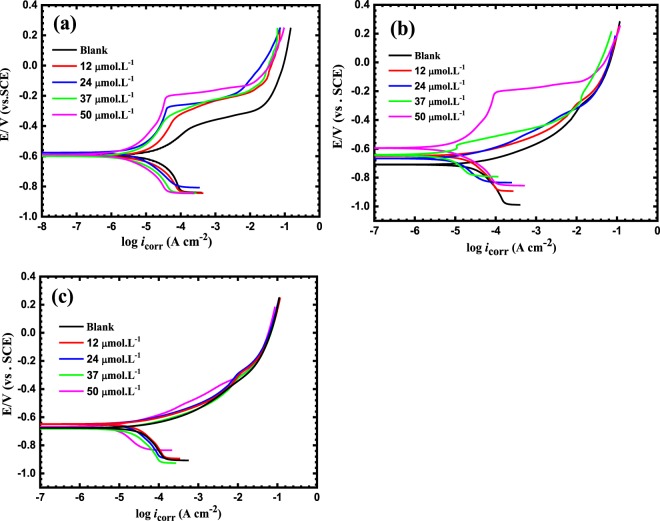
Polarization curves for reinforced steel in SCP solutions containing 3.5 wt.%NaCl and BTC inhibitor concentrations of 12, 24, 37, 50 μmol L^−1^ at pH values of (**a**) 12.5, (**b**) 10 and (**c**) 7.

The polarization curves for the reinforced steel in SCP solutions containing 3.5 wt.%NaCl and BTC inhibitor concentrations of 12, 24, 37, 50 μmol L^−1^ at pH values of 12.5, 10 and 7 are shown in Fig. 5.

The electrochemical corrosion factors such as as the corrosion free potential (*E*_corr_), pitting potential (*E*_pit_), corrosion current density (*i*_corr_), the polarization resistance, (*R*_p_), cathodic and anodic Tafel slopes (*b*_c_ and *b*_a_, respectively), the corrosion inhibition efficiency (*IE*%) and the surface coverage area (*θ*), are calculated from Fig. 5, and recorded in Table 2. Additionally, the passive potential window is calculated using the following formula:

4 $${E}_{pit}-{E}_{corr}$$

**Table 2:** The electrochemical factor derived from polarization plots of the reinforcing steel in SCP solutions including 3.5 wt.%NaCl and BTC inhibitor concentrations of 12, 24, 37, 50 μmol L^−1^ at pH values of 12.5, 10 and 7.

pH values	Inhibitor concertation (µmol L^−1^)	*i*_corr_ (µA cm^−2^)	*i*_pit_ (µA cm^−2^)	*E*_pit_ (mV) SCE	*E*_corr_ (mV) SCE	*E*_pit_-*E*_cor_ (mV) SCE	*B*c (V decade^−1^)	*B*a (V decade^−1^)	*R*_p_ Ω cm^2^	*IE*%	surface coverage (ϴ)
12.5	Blank	13	2.0 × 10^−4^	−0.41	−0.601	0.187	0.11	0.076	1502	—	—
	12	5.2	6.7 × 10^−5^	−0.34	−0.598	0.254	0.081	0.061	2888	56	0.56
	24	3.2	5.0 × 10^−5^	−0.33	−0.601	0.271	0.077	0.059	4590	76	0.76
	37	2.5	4.2 × 10^−5^	−0.28	−0.602	0.322	0.072	0.056	5471	81	0.81
	50	1.6	3.9 × 10^−5^	−0.21	−0.594	0.384	0.068	0.048	7636	88	0.88
10	Blank	20	—	—	−0.71	—	0.091	0.091	988	—	—
	12	15	—	—	−0.639	—	0.087	0.081	1214	22	0.22
	24	10	—	—	−0.665	—	0.075	0.079	1671	52	0.52
	37	8	1.23 × 10^−5^	—	−0.635	0.058	0.061	0.066	1721	63	0.63
	50	5	9.7 × 10^−5^	—	−0.585	0.376	0.058	0.058	2518	78	0.78
7	Blank	33	—	—	—	—	0.098	0.120	709	—	—
	12	28	—	—	—	—	0.087	0.110	755	15	0.15
	24	21	—	—	—	—	0.080	0.109	958	36	0.36
	37	14	—	—	—	—	0.071	0.111	1344	57	0.57
	50	9.5	—	—	—	—	0.116	0.091	2332	71	0.71

The corrosion inhibition efficiency (*IE*%), is calculated using the following formula^37^,

5 $$IE \% =(\frac{{i}_{1}-{i}_{2}}{{i}_{1}})\times 100$$

where *i*_1_ and *i*_2_ are the corrosion current densities of reinforcing steel in the absence and existence of the BTC corrosion inhibitor, respectively.

The surface coverage area (*θ*) is calculated utilizing Eq. 6^11^,

6 $$\theta =\frac{IE \% }{100\,}$$

and the polarization resistance (*R*_p_), was detected using the Stern–Geary equation^37^.

7 $${R}_{{\rm{p}}}=\frac{{b}_{{\rm{c}}}\,{b}_{{\rm{a}}}}{2.303\,{i}_{{\rm{corr}}}({b}_{{\rm{c}}}+{b}_{{\rm{a}}})}$$

The polarization curves show the breakdown of the passive film before and after the addition of BTC inhibitor at pH 12.5, see Table 2. However, increasing the inhibitor concentration shifts the pitting potentials (*E*_pit_), towards the more noble values indicating that the passive layer formed more stabilized by the presence of BTC inhibitor. Moreover, the passive potential window at pH 12.5 is 0.18, which increased to 0.25, 0.27, 0.32 and 0.38 by the addition of 12, 24, 37, 50 μmol L^−1^ of the BTC inhibitor, respectively. The attack of chloride species to the reinforcing steel surface in SCP can lead to loss of its passive layer if the concentration of the chloride species is adequately high. For reinforcing steel in concrete, the degree to which Cl¯ ions can damage the passive layer is related to the alkalinity of the environment. In chloride-free alkaline conditions, the passive layer on the mild steel breaks down at a potential of +560 mV SCE^38^. The highest attained *IE*% was 88% at 50 μmol L^−1^ of the BTC inhibitor at pH = 12.5. It can be seen that the values of *b*a and *b*c diminished upon the addition of the inhibitor indicating that BTC is a mixed type inhibitor. it is noteworthy to mention that the tabulated values of the corrosion density (*i*_corr_), shifts towards decreases with increasing the concertation of the BTC inhibitor. However, these values are not in accordance with reported articles in references^12,39^. In fact, the diffusion of chloride species (Cl^−^), in cementitious materials immersed in saline water is a difficult process, which includes numerous chemical and physical interactions. Cl^−^ ions can bound chemically or physically through the cement paste, thus reducing the segment of free Cl^−^ species that can diffuse easily in the concrete pore solutions. Furthermore, the internal electric field generated from the anions and cations will accelerate the ions that possess low diffusion coefficients and decelerate the ions that have high diffusion coefficients in order to keep the electro-neutrality status^40^.

### Adsorption isotherm

In order to understand and estimate the adsorption route of BTC on steel surface, different adsorption isotherms are checked using the measured data from the poanlarization plots e.g. Langmuir, Frumkin and Temkin isotherms using the following equations:

Langmuir

8 $$\frac{{C}_{{\rm{inh}}}}{\theta }=\frac{1}{{K}_{{\rm{ads}}}}+{C}_{{\rm{inh}}}$$

Frumkin

9 $$log\{C\times (\frac{{\rm{\theta }}}{1-{\rm{\theta }}})\}=2.303\,logK+2\alpha {\rm{\theta }}\,$$

**Figure 6:**
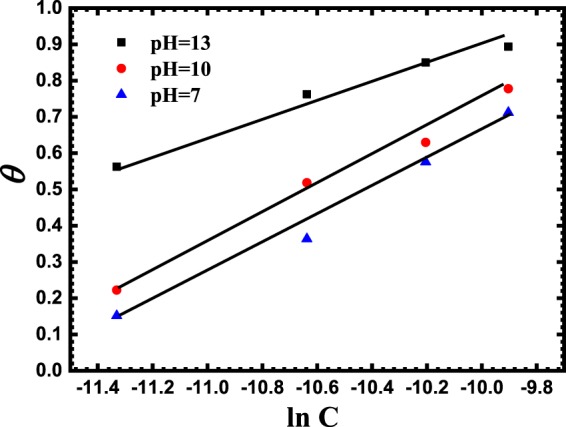
Temkin adsorption plots for reinforcing steel in SCP solution conatining 3.5 wt.% NaCl in the existence of 12, 24, 37, 50 μmol L^−1^ of BTC corrosion inhibitor at variable pH values of 12.5, 10, and 7.

Temkin

10 $$\exp (-2\alpha {\rm{\theta }})={K}_{{\rm{ads}}}C$$

where θ is the surface coverage of the reinforcement steel, *C* is the concentration of the BTC inhibitor species, *α* is the adsorbate interaction factor and *K*_ads_ is the adsorption– desorption equilibrium constant. The fitting outcomes showed that BTC inhibitor obeys Temkin isotherm, see Fig. 6.

After reorganizing Eq. (10), the following expression is attained:

11 $$\theta =\frac{1}{-2\alpha }lnc+\frac{1}{-2\alpha }ln{K}_{ads}$$

It can be deduced from Eq. (11) that both of the slope and intercept are calculated from 1/−2*α* and (1/−2*α)lnK*_*ads*_, respectively.

Knowing the *K*_ads_ values at various pH values, the standard Gibbs free energy change of adsorption (*∆G*°_ads_) are calculated using Eq. 12.

12 $${K}_{{\rm{ads}}}=\frac{1}{55.5}{e}^{-\frac{\Delta {G}_{{\rm{ads}}}^{{\rm{o}}}}{RT}}$$

Table 3 summarizes the values of the *α*, *K*_ads_ and *∆G*°_ads_. Values of *∆G*°_ads_ ≥ −20 kJ mol^−1^, showing a physisorption adsorption, while *∆G*°_ads_ ≤ −40 kJ mol^−1^ depicts chemisorption adsorption reactions. Consequently, the intermediate values of *∆G*°_ads_ shown in Table 3 (−32, −33 and −34 kJ mol^−1^), usually elucidate that chemi-physisorption of BTC inhibitor occurs on the reinforcing steel at different pH values of 12.5, 10 and 7, respectively. However, since there is no free electron pair existing in the molecular structure of the BTC inhibitor that can form coordinated covalent bond with the vacant d-orbitals in Fe (chemisorption), therefore it is more favorable that the adsorption mechanism is a strong physisorption ratherthan a chemi-physisorption one. Physical adsorption takes place rapidly because of weak bondings such as Van der Waal’s or electrostatic attractive forces between inhibitor species and metal surface, and is directly influenced by the electronegativity of the inhibitor compounds. The residence time for a physically adsorbed inhibitor is short, and its interaction with the steel surface is directly associated with the corrosion free potential of the metal corrosion with respect to the potential of zero charge.

**Table 3:** The calculated thermodynamic parameters derived from Temkin plot.

pH values	Slope	α	Intercept	*K*_ads_ × 10^4^, (L mole^−1^)	*∆G*°_ads,_ (kJ mol^−1^)
12.5	−0.33	0.17	−3	0.88	−32
10	−0.36	0.14	−3.5	1.6	−33
7	−0.39	0.13	−3.9	2.2	−34

**Figure 7:**
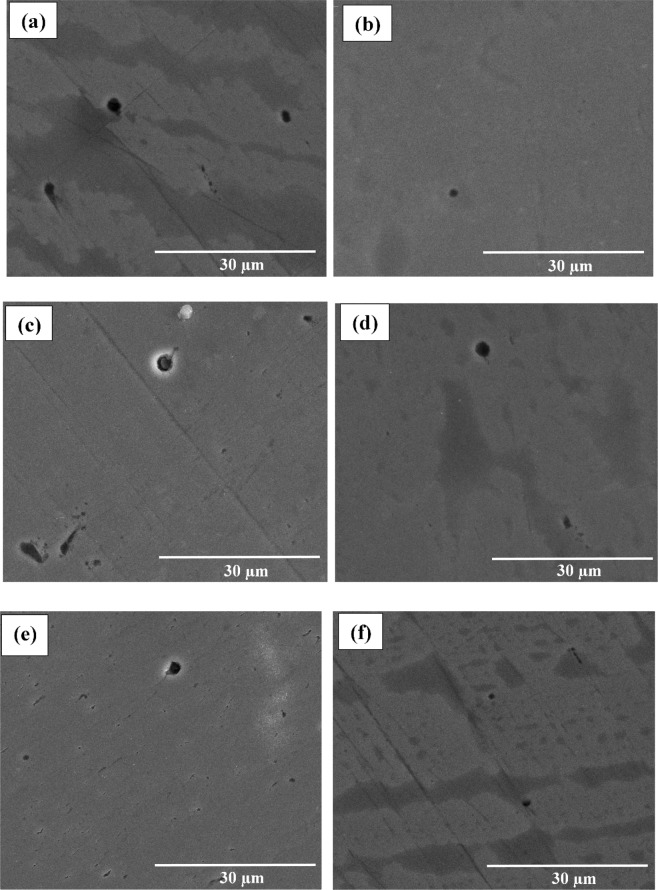
SEM images for the reinforcing steel (**a,c,e**) before and (**b,d,f**) after immersion in SCP solution containing 3.5 wt.% NaCl in existence of 50 μmol L^−1^ of BTC corrosion inhibitor at variable pH values of (**a,b**) 12.5, (**c,d**) 10, and (**e,f**) 7 for 24 h.

Figure 7 exhibits the SEM of the reinforcing steel coupons after immersion in SCP including 3.5 wt.% NaCl at different pH values of 12.5, 10, 7 in the existence and absence of 50 μmol L^−1^ of the BTC inhibitor for 24 h. It is clear that in case of the absence of the corrosion inhibitor, deep pits were formed and their number is suppressed as the pH of the medium increases. Nonetheless, in the existence of the corrosion inhibitor, the number and pits size are considerably reduced at the same pH values. Moreover, the pH values before and after immersion are measured. It is found that the pH values in the absence of the corrosion inhibitors are lowered from 12.5, 10 and 7 to 10, 8.7 and 5.7, respectively. However, in the existence of the BTC inhibitor the pH values slightly dropped from 12.5, 10 and 7 to 11.5, 9.3 and 6.3, respectively.

**Figure 8:**
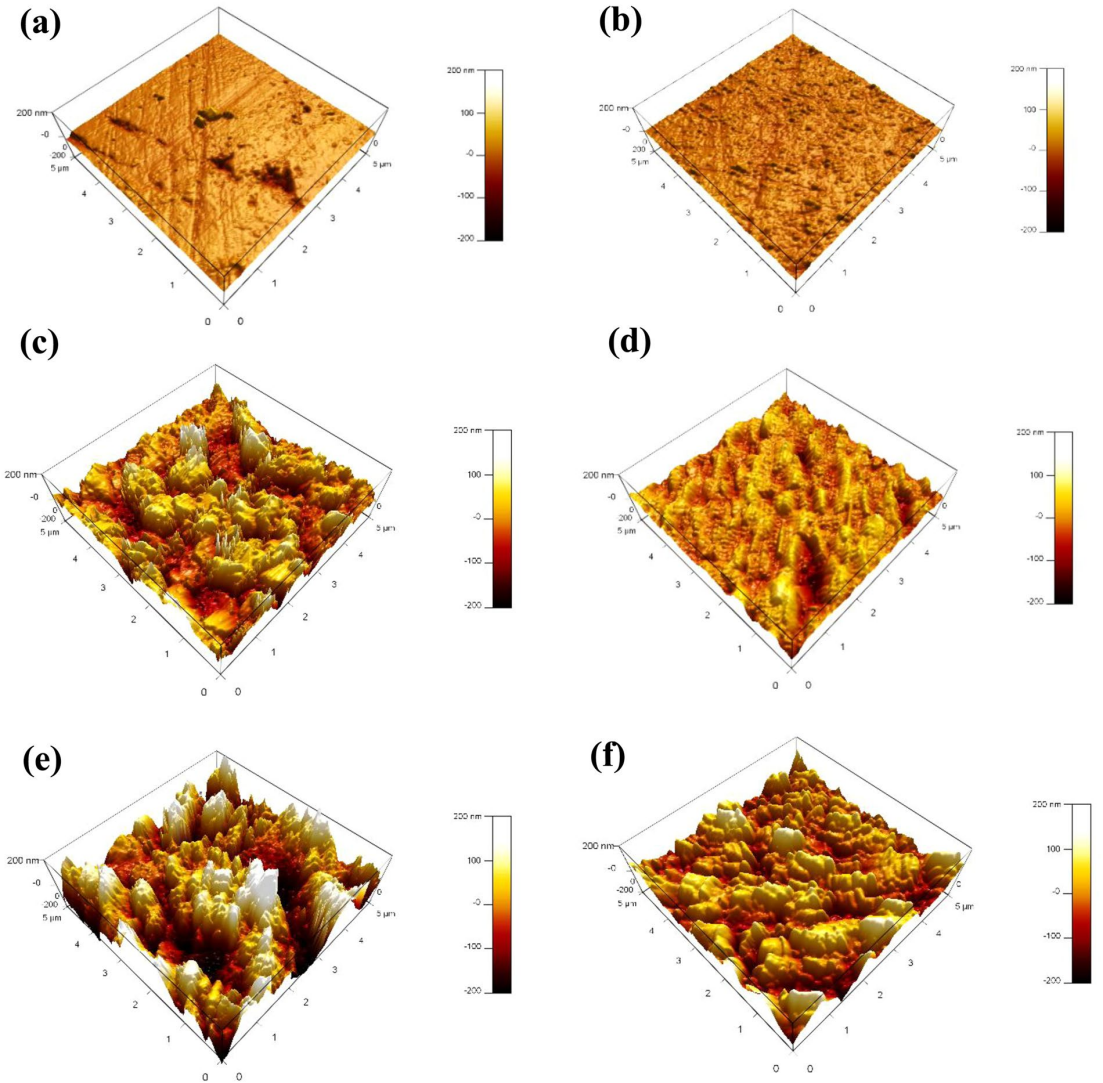
AFM images for the reinforcing steel surface (**a,c,e**) before and (**b,d,f**) after immersing the reinforcing steel for 24 h in SCP solution containing 3.5 wt.% NaCl in existence of 50 μmol L^−1^ of BTC corrosion inhibitor at variable pH values of (**a,b**) 12.5, (**c,d**) 10, and (**e,f**) 7.

Surface topography and surface roughness of the reinforcing steel are explored after immersion in 3.5 wt.%NaCl of variable pH values for 24 h in the existence and absence of 50 μmol L^−1^ of the BTC inhibitor using AFM, as depicted in Fig. 8. It is noted that the surface roughness (*R*_a_), escalates as the pH alleviates in the absence of the corrosion inhibitor, see Table 4. However, *R*_a_ is decreased significnatly in the existence of the BTC inhibitor in the deleterious medium signifying the construction of an adsorbed protective layer of BTC inhibitor on the metal surfaces, which retards the attack of the Cl^−^ species

**Table 4:** The surface roughness values (*R*_a_), of the reinforcing steel before and after addition of BTC inhibitor in saline water for 24 h at variable pH values.

pH	*R*_a_ in absence of BTC (nm)	*R*_a_ in presence of BTC (nm)
12.5	27	15
10	47	23
7	70	36

**Figure 9:**
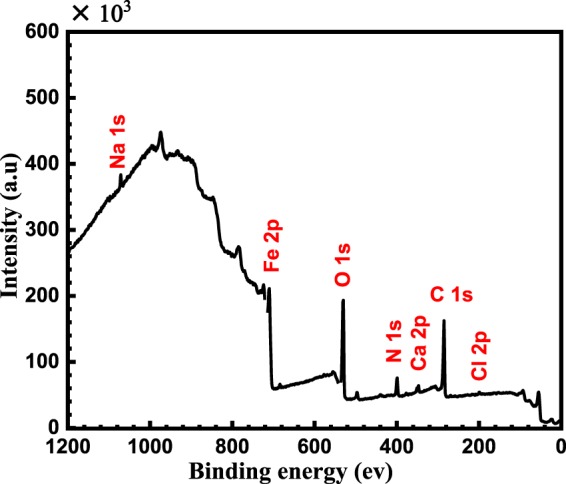
XPS survey scan composition of the mild steel after immersion for 24 h in SCP solution containing 3.5 wt.%NaCl in the existence of 50 μmol L^−1^ of BTC corrosion inhibitor at pH 12.5.

**Figure 10:**
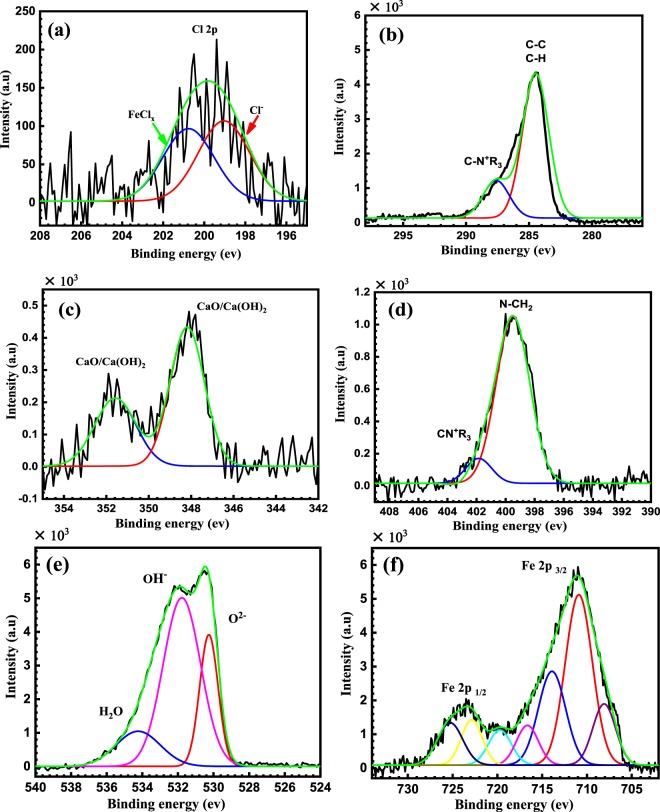
High resolution XPS spectrum of (**a**) Cl 2p, (**b**) C 1 s, (**c**) Ca 2p, (**d**) N 1 s (**e**) O 1 s and (**f**) Fe 2p after immersing the reinforcing steel for 24 h in SCP solution containing 3.5 wt.% NaCl in existence of 50 μmol L^−1^ of BTC corrosion inhibitor at pH Σ12.5.

The wide scan spectrum (Fig. 9) and the high resolution XPS spectra (Fig. 10) are obtained after immersing the reinforcing steel for 24 h in SCP including 3.5 wt.% NaCl in the existence of 50 μmol L^−1^ of BTC corrosion inhibitor at pH 12.5.

The Cl 2p spectrum is deconvoluted into two components at BE of 199.1 and 200.7 eV which are attributed to Cl^−^ and FeCl_x_, respectively^41^, see Fig. 10a. It is noteworthy that the peak intensity of Cl^−^ species is low; indicating that the adsorbed BTC inhibitor lowers the adsorption affinity of the Cl^−^ ions to the metal surface which subsequently lessens the corrosion rate. The adsorption of the BTC inhibitor on the reinforcing steel surface is further confirmed by the analysis of C spectra, which showed the presence of CN^+^-R_3_ and (C-C & C-H) at BE of 284.5 and 287.7 eV, respectively^42,43^, see Fig. 10b. However, Ca 2p spectrum shown in Fig. 10c is decomposed into two bands at 347.8 eV and 351.5 eV that are credited to Ca 2P_3/2_ and 2P_1/2_ respectively, of CaO/Ca(OH)_2_^8^. Figure 10d shows the appearance of N 1 s peaks at 399.4 and 402.5 eV, characteristics of N-CH_2_ and N^+^ quaternary nitrogen, respectively^43-46^. This approves the adsorption of the BTC inhibitor on the reinforcing steel surface. There is no peak for C–N–Fe bonding was observed at 397.7–398.6 eV, suggesting that BTC inhibitor was adsorbed on the reinforcing steel through physisorption^47^. The XPS spectrum of O 1 s in Fig. 10e, exhibits three peaks at 530.3, 531.7 and 534.2 eV, that are credited to O^2−^ of iron oxides, OH^−^ of hydrous iron oxides (FeOOH), and H_2_O, respectively^43,46^. On the other hand, Fe 2p spectra in Fig. 10f is deconvoluted into six peaks. In fact, the interpretation of Fe 2p spectra is a complex owing to the existence of iron (Fe), in variable oxidation states of Fe°, Fe^2+^, Fe^3+^, and satellites of Fe^3+^ species. The (Fe 2p_3/2_), XPS spectra at high resolution involves four bands at 707.1 eV that is related to the metallic iron, 710.9 eV for Fe^3+^ of Fe_2_O_3_/ FeOOH and 713.9 eV, which could be attributed to a mixture of (Fe^2+^ & Fe^3+^), in different forms of iron (II) oxide (FeO), iron (II) hydroxide Fe(OH)_2_, iron (III) hydroxide Fe(OH)_3_, FeOOH, iron (III) oxide (Fe_2_O_3_), and magnetite (Fe_3_O_4_)^48^. The shake up phenomenon found at 716.6 and 719.8 eV is ascribed to Fe^2+^ and Fe^3+^, respectively. The spectra of the Fe 2p_1/2_ peaks at BE of 722.7 and 725.3 eV can be ascribed to Fe_2_O_3_ and FeO(OH), respectively^49^.

### Mechanical properties

Although corrosion inhibitors can protect steel against corrosion, however it can badly affect its mechanical features^50^. Therefore, the mechanical characteristics of cured mortars are investigated in the presence and absence of a 20 ppm (50 μmol L^−1^), of the BTC inhibitor. This is achieved through measuring the flexural and compressive strength of cement, see Table 5. It can be observed that there is almost no change in the mechanical properties of the concrete after the addition of the inhibitor, which is attributed to the low concentration of the BTC.

**Table 5:** Flexural and compressive strength results of the as-prepared cured mortars after different curing times.

Exposure time (days)	Flexural strength Maximum Bending Stress at Break, (MPa)		Compression strength (N mm^−2^)	
	Blank	BTC	Blank	BTC
7	5.9	5.6	42.2	39.1
14	6.3	6.3	44. 2	44.2
21	6.5	6.2	47.31	47.1
28	6.6	6.4	47.4	47.3

## Conclusions

A new BTC corrosion inhibitor for reinforcing steel is investigated in 3.5 wt.% NaCl at different pH values. Taflel plots indicated that BTC is a mixed type inhibitor. The BTC inhibitor showed a corrosion inhibition efficiency (IE%) of 88, 78 and 71% in 3.5 wt.% NaCl using 50 μmol L^−1^ at pH values of 12.5, 10 and 7, respectively, which is effective if carbonation of the concrete happens and the pH of the concrete is lowered. Based on the adsorption isotherm calculations, BTC inhibitor showed the best fitting with Temkin isotherm. XPS results illustrate that BTC inhibitor is physisorbed on the reinforcing steel surface, which is matching with the *∆G*°_ads_ calculations. The surface roughness of the metal surface is significantly decreased upon the addition of BTC inhibitor as shown in the AFM results confirming a high corrosion inhibition efficiency of BTC. Additionally, no change in the mechanical features of concrete is observed upon the addition of BTC inhibitor, which allows using it in concrete without any reservation.

## Data Availability

The raw/processed data required to reproduce these findings could not be shared at this time due to time limitations. However, it will be available on request.

## References

[1] Ormellese M, Lazzari L, Goidanich S, Fumagalli G, Brenna A (2009). A study of organic substances as inhibitors for chloride-induced corrosion in concrete. Corrosion Science.

[2] Diamond S (1986). Chloride concentrations in concrete pore solutions resulting from calcium and sodium chloride admixtures. Cement, concrete and aggregates.

[3] Enevoldsen JN, Hansson CM, Hope BB (1994). The influence of internal relative humidity on the rate of corrosion of steel embedded in concrete and mortar. Cement and concrete research.

[4] Hussain SE, Al-Musallam A, Al-Gahtani AS (1995). Factors affecting threshold chloride for reinforcement corrosion in concrete. Cement and Concrete Research.

[5] Moreno M, Morris W, Alvarez MG, Duffó GS (2004). Corrosion of reinforcing steel in simulated concrete pore solutions: effect of carbonation and chloride content. Corrosion Science.

[6] Ghods P, Isgor OB, McRae G, Miller T (2009). The effect of concrete pore solution composition on the quality of passive oxide films on black steel reinforcement. Cement and Concrete Composites.

[7] Liu Y (2019). Effect of ginger extract as green inhibitor on chloride-induced corrosion of carbon steel in simulated concrete pore solutions. Journal of Cleaner Production.

[8] Wang Y, Zuo Y, Zhao X, Zha S (2016). The adsorption and inhibition effect of calcium lignosulfonate on Q235 carbon steel in simulated concrete pore solution. Applied Surface Science.

[9] El Haleem SMA, El Wanees SA, El Aal EEA, Diab A (2010). Environmental factors affecting the corrosion behavior of reinforcing steel. IV. Variation in the pitting corrosion current in relation to the concentration of the aggressive and the inhibitive anions. Corrosion Science.

[10] El Wanees SA, Radwan AB, Alsharif MA, El Haleem SMA (2017). Initiation and inhibition of pitting corrosion on reinforcing steel under natural corrosion conditions. Materials Chemistry and Physics.

[11] Yang D (2019). Functionalization of citric acid-based carbon dots by imidazole toward novel green corrosion inhibitor for carbon steel. Journal of Cleaner Production.

[12] Goñi S, Andrade C (1990). Synthetic concrete pore solution chemistry and rebar corrosion rate in the presence of chlorides. Cement and Concrete Research.

[13] Andrade, C., Alonso, M. & Gonzalez, J. In *An Initial Effort to Use the Corrosion Rate Measurements for Estimating Rebar Durability* (1990).

[14] Andrade, C. & Alonso, M. In *Values of Corrosion Rate of Steel in Concrete to Predict Service Life of Concrete Structures* (1994).

[15] El Haleem SMA, El Wanees SA, Bahgat A (2014). Environmental factors affecting the corrosion behaviour of reinforcing steel. VI. Benzotriazole and its derivatives as corrosion inhibitors of steel. Corrosion Science.

[16] Oranowska H, Szklarska-Smialowska Z (1981). An electrochemical and ellipsometric investigation of surface films grown on iron in saturated calcium hydroxide solutions with or without chloride ions. Corrosion Science.

[17] Eyu DG, Esah H, Chukwuekezie C, Idris J, Mohammad I (2013). Effect of green inhibitor on the corrosion behaviour of reinforced carbon steel in concrete. ARON Journal of engineering and Applied sciences.

[18] Jiang S (2017). Deoxyribonucleic acid as an inhibitor for chloride-induced corrosion of reinforcing steel in simulated concrete pore solutions. Construction and Building Materials.

[19] Zhang Z, Ba H, Wu Z, Zhu Y (2019). The inhibition mechanism of maize gluten meal extract as green inhibitor for steel in concrete via experimental and theoretical elucidation. Construction and Building Materials.

[20] Basha Nusrath Unnisa C (2018). Linear polyesters as effective corrosion inhibitors for steel rebars in chloride induced alkaline medium – An electrochemical approach. Construction and Building Materials.

[21] Fazayel AS, Khorasani M, Sarabi AA (2018). The effect of functionalized polycarboxylate structures as corrosion inhibitors in a simulated concrete pore solution. Applied Surface Science.

[22] Shanmugapriya S, Prabhakar P, Rajendran S (2018). Corrosion Resistance Property of Mild Steel in Simulated Concrete Pore Solution Prepared in Well Water by Using an Aqueous Extract of Turmeric. Materials Today: Proceedings.

[23] Anitha R (2019). Implications of eco-addition inhibitor to mitigate corrosion in reinforced steel embedded in concrete. Construction and Building Materials.

[24] Wang Y, Zuo Y (2017). The adsorption and inhibition behavior of two organic inhibitors for carbon steel in simulated concrete pore solution. Corrosion Science.

[25] Cao F, Wei J, Dong J, Ke W (2015). The corrosion inhibition effect of phytic acid on 20SiMn steel in simulated carbonated concrete pore solution. Corrosion Science.

[26] Asaad MA (2018). Enhanced corrosion resistance of reinforced concrete: Role of emerging eco-friendly Elaeis guineensis/silver nanoparticles inhibitor. Construction and Building Materials.

[27] Cameron DM (2013). Confirmation of *in vitro* and clinical safety assessment of behentrimonium chloride-containing leave-on body lotions using post-marketing adverse event data. Toxicology in Vitro.

[28] Ai Z (2016). Passive behaviour of alloy corrosion-resistant steel Cr10Mo1 in simulating concrete pore solutions with different pH. Applied Surface Science.

[29] ASTM C348-18, Standard Test Method for Flexural Strength of Hydraulic-Cement Mortars, *ASTM International, West Conshohocken, PA*, doi:www.astm.org (2018).

[30] ASTM C109/C109M-16a, Standard Test Method for Compressive Strength of Hydraulic Cement Mortars (Using 2-in or [50-mm] Cube Specimens), *ASTM International, West Conshohocken, PA*, doi:www.astm.org (2018).

[31] Poursaee A, Hansson CM (2007). Reinforcing steel passivation in mortar and pore solution. Cement and Concrete Research.

[32] Ghantous RM, Poyet S, L’Hostis V, Tran N-C, François R (2017). Effect of crack openings on carbonation-induced corrosion. Cement and Concrete Research.

[33] Wan K, Li L, Sun W (2013). Solid–liquid equilibrium curve of calcium in 6mol/L ammonium nitrate solution. Cement and Concrete Research.

[34] Macdonald JR (1987). Impedance spectroscopy and its use in analyzing the steady-state AC response of solid and liquid electrolytes. Journal of Electroanalytical Chemistry and Interfacial Electrochemistry.

[35] J. R. & MacDonald. Impedance Spectroscopy Emphasizing Solid Materials and Systems. *John Wiley & Sons, New York* (1987).

[36] Frankel GS (1998). Pitting Corrosion of Metals: A Review of the Critical Factors. Journal of The Electrochemical Society.

[37] Ahamad I, Prasad R, Quraishi MA (2010). Adsorption and inhibitive properties of some new Mannich bases of Isatin derivatives on corrosion of mild steel in acidic media. Corrosion Science.

[38] Martin, F. & Olek, J. The Nature of Passivity of Reinforcing Steel. *ASCE Materials Congress*, Washington, D.C (1996).

[39] Stefanoni M, Angst U, Elsener B (2018). Corrosion rate of carbon steel in carbonated concrete – A critical review. Cement and Concrete Research.

[40] Shi X, Xie N, Fortune K, Gong J (2012). Durability of steel reinforced concrete in chloride environments: An overview. Construction and Building Materials.

[41] Zhou X, Yang H, Wang F (2012). Investigation on the inhibition behavior of a pentaerythritol glycoside for carbon steel in 3.5% NaCl saturated Ca(OH)2 solution. Corrosion Science.

[42] Cao W, Wang Z, Zeng Q, Shen C (2016). 13C NMR and XPS characterization of anion adsorbent with quaternary ammonium groups prepared from rice straw, corn stalk and sugarcane bagasse. Applied Surface Science.

[43] Swift A, Paul AJ, Vickerman JC (1993). Investigation of the surface activity of corrosion inhibitors by XPS and time-of-flight SIMS. Surface and Interface Analysis.

[44] Santos AR, Blundell RK, Licence P (2015). XPS of guanidinium ionic liquids: a comparison of charge distribution in nitrogenous cations. Physical Chemistry Chemical Physics.

[45] Park JS (2012). A ZnO/N-doped carbon nanotube nanocomposite charge transport layer for high performance optoelectronics. Journal of Materials Chemistry.

[46] Tourabi M, Nohair K, Traisnel M, Jama C, Bentiss F (2013). Electrochemical and XPS studies of the corrosion inhibition of carbon steel in hydrochloric acid pickling solutions by 3,5-bis(2-thienylmethyl)-4-amino-1,2,4-triazole. Corrosion Science.

[47] Finšgar M, Fassbender S, Hirth S, Milošev I (2009). Electrochemical and XPS study of polyethyleneimines of different molecular sizes as corrosion inhibitors for AISI 430 stainless steel in near-neutral chloride media. Materials Chemistry and Physics.

[48] Solomon MM, Umoren SA, Obot IB, Sorour AA, Gerengi H (2018). Exploration of Dextran for Application as Corrosion Inhibitor for Steel in Strong Acid Environment: Effect of Molecular Weight, Modification, and Temperature on Efficiency. ACS Applied Materials & Interfaces.

[49] Grosvenor AP, Kobe BA, Biesinger MC, McIntyre NS (2004). Investigation of multiplet splitting of Fe 2p XPS spectra and bonding in iron compounds. Surface and Interface Analysis.

[50] De Schutter G, Luo L (2004). Effect of corrosion inhibiting admixtures on concrete properties. Construction and Building Materials.

